# Urgent appeal to minimize community health impacts of COVID-19

**DOI:** 10.4178/epih.e2020021

**Published:** 2020-04-29

**Let us overcome COVID-19 outbreak by the citizen-led defense.**

Despite our desire to block its community spread and total blockade from the authorities, the community spread of coronavirus disease 2019 (COVID-19) has come to pass. However, we need not fear it. Fortunately, as COVID-19 has been identified case after case, more sophisticated responses have become possible. COVID-19 is a novel infectious disease that is now spreading worldwide, but human history has never been free from the challenge of new infectious diseases. We have overcome severe acute respiratory syndrome (SARS) and Middle East respiratory syndrome (MERS), which are far more deadly compared to COVID-19 and have developed the swine flu vaccine to control the H1N1 virus, which is far more contagious compared to COVID-19.

As of February 20, 2020, the fatality rate of COVID-19 is 3.3% in Hubei but less than 1% outside of the Hubei province (0.7%) or in countries outside of mainland China (0.9%). This is higher than the 0.05% fatality rate of H1N1 influenza but significantly lower than the 10% fatality rate of SARS or the 30% fatality rate of MERS. However, the community spread of COVID-19 is very fast and widespread because of its mild initial symptoms, such as common cold, and the high levels of virus shed during this period. This can cause serious consequences to vulnerable groups, such as the elderly patients and patients with chronic diseases. According to the reports in China, the elderly people aged 60 years or older account for 30% of all cases and over 80% of fatal cases.

It is virtually impossible to completely block the community spread of new, highly contagious viral infections. By far, more than 25 countries have reported confirmed cases, and community spread has reached nearby countries, including Hong Kong, Japan, and Singapore. Therefore, it is now time to move toward a mitigation strategy (second prevention) that delays the community spread and minimizes health damages from the blockade-centered containment strategy (primary prevention), confirming cases and ensuring contact isolation.

In other words, the goals and strategies of the quarantine should be modified to meet the changing situation of community spread. In this regard, “Pan-Academic Action Committee on COVID-19” would like to offer several actions to the government and fellow citizens.

To government,

First, please review necessary measures for an effective damage minimization (mitigation) strategy shifting from containment strategy. Particularly, take immediate action to strengthen the integrated defense activity system at the community level.

Second, please urgently establish an emergency medical delivery system to respond to COVID-19 spread to local communities. It is also important to ensure that the normal medical activity at primary care institutions not be affected. In the current situation, it is unlikely to expect the effectiveness of existing strategies, which focus on contact management and identifying sources of exposures. Therefore, establishing a concrete medical system with strong mobility restrictions is necessary. Please designate and support medical institutions dedicated to fever screening for respiratory syndrome and COVID-19 treatment hospitals to ensure that high-risk patients can be treated safely at general medical institutions. Additionally, please establish specific and effective health protection measures to prevent gaps in medical service delivery for the emergency, elderly, and chronic patients.

Third, please strengthen crisis communication activities to make accurate judgments and increase the trust and confidence of the public in the government through quick sharing of information with the public. To this end, create a multi-faceted communication channel for regular dialogue with authorities, infectious disease specialists, and community members. By doing so, encourage residents to actively participate in preventing the spread to their communities.

Fourth, please enable children, students, and employees with fever and respiratory symptoms to spend sick leave without a medical certificate. The government and businesses should make adjustments to help parents avoid disadvantages when taking sick leave for a sick child.

Finally, please recognize that human rights equal protection and take measures to ensure that the vulnerable groups, foreigners, and differently abled be able to receive COVID-19 treatment.

To fellow citizens,

Another crisis caused by the novel virus has been seriously discouraging the normal economic activity of our society and disturbing our daily lives. However, if everyone works together, we can overcome this crisis. To this end, the medical staff and local defense personnel are doing their best. We would like to ask you to trust and support them while offering several actions.

First, please wash your hands thoroughly and often with soap for at least 30 seconds and thoroughly maintain your hygiene by covering your mouth and nose with a tissue or sleeve when coughing or sneezing.

Second, if you have mild respiratory symptoms, such as fever, cough or sore throat, nasal congestion or runny nose, please refrain from going out and observe the symptoms while taking 4–5 days of general cold medicine. See your doctor if you have a fever of 38°C or higher or your symptoms worsen or continue. If symptoms persist, visit your nearest screening clinic or call your local health department or 1339. People with mild symptoms do not have to go to a large hospital, as large hospitals should be allowed to focus on treating seriously ill patients. Please refrain from going outside for more than 5 days after the onset of symptoms, as spreading can occur early even with mild symptoms. Fully assure the person to take absence or sick leave from school or work.

Third, people with chronic conditions or those over 65 years of age are more vulnerable to infections. Please avoid large public gatherings or places with large crowds. Wear a mask when you go out.

Fourth, we particularly ask students, parents, and teachers that if students have a fever or respiratory symptoms, please refrain from sending them to school. While the weather is still cold, check and prepare the facility for students to wash their hands frequently with soap under running water.

Finally, please actively follow the actions of medical staff and defense authorities to prevent the spread of COVID-19 in the local community. Even with a small deviation that is being repeated during the community outbreak, it is hard to overcome this crisis with any defense network.

Together, let us all overcome this challenge by utilizing all our social resources and capacities in a calm and unified way!

February 22, 2020

Pan-Academic Action Committee on COVID-19

The Korean Society of Infectious Diseases, The Korean Academy of Tuberculosis and Respiratory Diseases, The Korean Society of Pediatric Infectious Diseases, The Korean Society for Preventive Medicine, The Korean Society of Emergency Medicine, Korean Society for Healthcare-associated Infection Control and Prevention, The Korean Society of Critical Care Medicine, Korean Society for Antimicrobial Therapy, Korean Society of Epidemiology

