# Supplementary material for: Urgent appeal to minimize community health impacts of COVID-19
Source: Epidemiol Health. 2020 Apr 29;42:e2020021. doi: 10.4178/epih.e2020021 (PMC7285419; doi:10.4178/epih.e2020021)
Supplement: Supplementary file 1 [file epih-42-e2020021-suppl.pdf]

Volume: 42, Article ID: e2020021

https://doi.org/10.4178/epih.e2020021

「코로나 19 지역사회 건강피해 최소화」를 위한 긴급 호소문

이제 국민이 주도하는 방역으로 코로나19를 극복합시다.

우리나라는 1 월 20 일 첫 코로나 19 확진자가 보고된 이래 철저한 역학조사와 광범 위한 접촉자 관리를 통해 추가 전파를 비교적 잘 막아 왔습니다. 그러나 2 월 18 일 확 진된 31 번째 환자를 통해 종교단체 집회에 참석한 많은 사람이 이미 감염된 것을 확 인하게 되었습니다. 이러한 집단 유행이 대구와 경북에서 시작되어 전국으로 확산되 면서 지역사회에서 감당하기 어려운 수준으로 전개되고 있습니다.

'범학계 코로나 19 대책위원회'는 이러한 갑작스러운 상황 전개를 매우 심각한 공 중보건 위기 상황으로 인식하고 있고, 이러한 위협에 효과적으로 대응하기 위한 구체 적인 방역대책이 적시에 마련되어야 한다고 생각합니다. 이를 통해, 제한된 의료자원 으로 지역주민과 중증 확진자의 건강피해를 최소화하기 위해서는 바이러스의 지역사 회 확산 속도를 최대한 지연시켜야 합니다. 이를 위한 가장 핵심적인 방역대책은 **국민 모두가 참여하여 사람들 간의 접촉을 최소화하는 것입니다.** 이는 2 월 28 일 발표된 대한의사협회의 대국민 권고문에서도 "불필요한 접촉을 최대한 줄이자"라고 강조한 바 있습니다.

코로나19는 젊고 건강한 사람에게는 대부분 가벼운 증상으로 끝나지만, 노인과 만 성질환자에게는 위중한 결과를 초래할 수 있습니다. 코로나19는 특히 증상 초기 또는 증상을 느끼지 못하는 초기에 다량의 바이러스를 배출하여 지역사회 전파 위험이 어 느 감염병보다 높은 특징이 있습니다. 따라서 이 감염병은 우리나라뿐 아니라 전 세 계적인 대유행으로 확대될 것으로 예측되고 있습니다. 코로나19 **감염 유행의 건강피 해를 최소화하기 위해 가장 중요한 것은 국민들이 방역의 대상이 아닌 주체로서 참여 하는 것**입니다.

학교는 3월 9일로 개학을 연기하였고, 일부 민간기업에서는 재택근무 도입과 근무 시간 유연제 등을 통하여 사람들의 접촉을 최소화하기 위한 노력을 하고

있습니다. 그러나 **전파력이 높은 감염병 유행 시 일부 집단의 참여만으로는 감염병 예방의 효과 가 크지 않다**는 것이 이미 다수의 연구에서 보고되고 있습니다. 코로나19로 인한 지 역사회 감염 확산을 효과적으로 지연, 억제하기 위해서는 **더 강력한 전국적인 사회적 접촉 감소를 위한 노력이 필요**합니다.

지금부터 2 주가 중요합니다. 이에 '범학계 코로나 19 대책위원회'는 정부와 국민 여러분에게 다음을 부탁드립니다.

정부는,

첫째, **사람들의 접촉을 최소화하기 위한 재택근무, 근무시간 유연제, 대면 서비스 최 소화, 집단행사나 모임 제한 등에 공공뿐 아니라 민간기업도 모두 동참할 수 있도록 적극적인 방안을 신속히 시행**해 주시기 바랍니다.

둘째, 보건의료, 정치경제, 종교, 문화체육, 노동 분야 등에서 **우리 사회를 선도하는 주요 단체들과 긴급 소통하여 코로나19 예방을 위한 사회적 합의를 도출**해 주시기 바 랍니다.

셋째, 노인, 만성질환자, 각종 시설 입소자 등 **코로나19 감염에 취약한 집단과 코로나 19로 생활이 어려워진 저소득계층을 점검하고 이들에게 필요한 마스크 등의 방역물품 과 긴급생활지원을 우선적으로 해 주시기 바랍니다**. 아울러, 코로나19의 전국 확산으 로 경제적으로 큰 타격을 받고 있는 동네 상권 자영업자에 대한 특단의 지원 조치를 취해 주시길 바랍니다.

넷째, **코로나 19 의 장기적 유행과 대규모 유행 모두를 고려한 의료시설, 공공시설을 확보하고 보건의료물품 수급 계획을 세워 지역보건의료체계가 유지될 수 있도록** 해주 시기 바랍니다.

다섯째, **중앙정부와 지자체는 질병관리본부의 정책을 적극 지원하고 의료진의 안전과 사기진작을 위한 최선의 지원책을 강구**해 주시기 바랍니다.

Volume: 42, Article ID: e2020021

https://doi.org/10.4178/epih.e2020021

국민 여러분께 부탁드립니다.

첫째, 가능한 모든 모임과 외출을 자제하시고 사람들 간의 접촉을 최소화해 주시기 바랍니다. 집안에서 지낼 때 적절한 신체활동과 운동, 균형 있고 위생적인 식생활을 유지하시길 바랍니다. **실내 환기를 자주 해 주시기 바랍니다**.

둘째, **손을 자주 비누로 씻거나 손소독제를 사용하십시오**. 손을 통해 감염될 수 있기 때문에 씻지 않은 손으로 얼굴을 만지지 않도록 주의하시기 바랍니다. 손이 닿는 물 건은 소독제로 닦아 주시기 바랍니다.

Volume: 42, Article ID: e2020021

https://doi.org/10.4178/epih.e2020021

셋째, 감기증상이 있으면 3-4일 집에서 쉬면서 경과를 관찰한 후에 진료를 받으시기

바랍니다. 특히 **호흡기 증상으로 병원을 찾으실 때는 안심병원을 방문**해 주시기 바랍 니다. **만성질환으로 평소에 드시는 약이 있으신 분은 다니시던 병의원을 방문하시기 전에 전화로 상담**을 받으시기 바랍니다. 가족이 대신 약을 받아오셔도 됩니다.

넷째, 모두가 코로나19로 민감해지고 힘든 시기입니다. **서로 격려하고 응원하시면서 이 시기를 함께 극복해 나가도록 힘을 모아 주시기 바랍니다. 개개인의 건강 지키기 가 곧 우리 사회 건강 지키기의 초석이 됩니다.**

다섯째, 코로나19에 대한 막연한 두려움, 소문 및 잘못된 정보, 그리고 특정 집단에 대한 **낙인과 차별은 신속하고 효율적인 방역 대응을 어렵게 합니다.** 더 이상의 전파 를 막고 우리 공동체의 우려를 완화하기 위해서 **지역 주민 서로 신뢰와 연대감을 강 화**해 주시길 바랍니다.

끝으로, 코로나19 치료와 예방에 혼신의 힘을 다하고 있는 **의료진과 방역당국의 조치 에 적극 따라 주시고 응원해 주시기 바랍니다.**

한마음으로, 차분하게, 전 사회역량을
모아 이 어려움을 극복해 나갑시다 !!

2020. 2. 29

범학계 코로나 19
대책위원회

대한감염학회 대한감염관리간호사회 대한결핵및호흡기학회 대한소아감염학회 대한예방의학회 대한응급의학회 대한의료관련감염관리학회 대한임상미생물학회 대한중환자의학회 대한항균요법학회 한국역학회

Volume: 42, Article ID: e2020021

https://doi.org/10.4178/epih.e2020021

*** 사회적 접촉을 최소화하기 위한 행동 요령 예시**

- 기업은 재택근무를 확대하고, 대면 서비스는 가능한 모두 전화나 온라인 서비스로 대체한다.
- 학교와 학원 등은 개학을 연기하고 온라인 수업으로 대체한다.
- 다중이용시설(박물관, 극장, 영화관, 도서관 등)을 휴관한다.
- 대규모 행사, 집회, 종교 활동을 중단한다.
- 필수 업종, 필수 공공서비스를 제외한 모든 상업 활동을 중단한다.
- 사람들 간의 접촉이 있는 스포츠, 여가 활동 등을 중단한다.
- 국내외 출장, 교육연수, 해외여행을 연기한다.
- 지역 간 이동을 자제한다.
- 병의원, 시설 등의 방문을 자제한다.
